# The relationship between vitamin D levels, comprehensive geriatric assessment and anthropometric measurements

**DOI:** 10.1371/journal.pone.0329649

**Published:** 2025-08-07

**Authors:** Meryem Cakir, Yasemin Ozkaya, Nafiye Ebru Terzi, Olgu Aygun, Halime Seda Kucukerdem, Elif Saki

**Affiliations:** 1 Izmir City Hospital, Departmant of Family Medicine, İzmir, Turkey; 2 Department of Family Medicine, Health Science University, Izmir Bozyaka Education and Research Hospital, Izmir, Turkey; City College of New York, UNITED STATES OF AMERICA

## Abstract

**Introduction:**

It is well-established that vitamin D deficiency increases with age. This study aims to investigate the relationship between vitamin D levels, comprehensive geriatric assessment and anthropometric measurements in elderly individuals.

**Methods:**

In this retrospective cross-sectional analytical study, data from patients aged 65–100 registered with İzmir City Hospital Home Care Services were analyzed. The patients’ vitamin D levels were compared with their sociodemographic and clinical data, anthropometric measurements (BMI, ABSI, body fat percentage), and geriatric assessment results (activities of daily living, nutrition, mental status, depression, frailty, and sarcopenia). Data were analyzed using SPSS 26.0 Statistics program.

**Results:**

This retrospective cross-sectional study included 439 elderly individuals aged 65–100 years receiving home healthcare services. In univariate analysis, no significant associations were found between vitamin D and other variables, except for hypertension (p = 0.049). ABSI values were higher in individuals vitamin D level with severe deficient and deficient compared to other groups, while BMI levels were found to be higher in the vitamin D deficient group (p < 0.001, p < 0.001, respectively). Additionally, the severe deficiency group had a higher frequency of pressure ulcers and lower Barthel Index scores (p = 0.033, p = 0.026, respectively). Correlation analysis revealed a significant negative relationship between vitamin D levels and ABSI (r = −0.184, p < 0.05) and the presence of pressure ulcers (r = −0.113, p < 0.05). Regression analysis confirmed these negative associations with ABSI (β = −0.179, p < 0.05) and pressure ulcers (β = −0.113, p < 0.05). Hypertension and BMI lost their significance in correlation and regression analyses.

**Conclusion:**

Lower vitamin D levels were associated with higher ABSI values and a greater frequency of pressure ulcers. ABSI may be useful in predicting vitamin D deficiency, and monitoring vitamin D levels in patients with pressure ulcers is recommended.

## Introduction

Vitamin D is a fat-soluble vitamin synthesized by the body upon exposure to sunlight [1]. Additionally, it can be obtained exogenously through the consumption of foods such as fatty fish, mushrooms, and egg yolk. While vitamin D is primarily essential for maintaining calcium and phosphate balance, which is crucial for bone health, it also plays a significant role in overall cellular functions throughout the body. The literature suggests that vitamin D may have protective effects against various diseases, including osteoporosis, hypertension, cancer, and obesity [1]. According to the World Health Organization (WHO), the prevalence of vitamin D deficiency varies between 30% and 90%, depending on the type of study, country, age group, and analytical method used [2].

Vitamin D deficiency is known to increase with age [3]. This is attributed to reduced mobility leading to decreased sun exposure, a decline in the skin’s ability to synthesize vitamin D, and age-related deterioration in kidney function. Studies indicate that vitamin D production decreases by approximately 13% per decade, and by the age of 70, individuals produce only half as much vitamin D as those in their 20s [3]. In older adults, this deficiency has been associated with impairments in mental status, an increased risk of depression, and a higher susceptibility to frailty, all of which are critical factors in geriatric assessment [2,3].

Most studies examining the relationship between vitamin D levels and anthropometric measurements have primarily focused on body mass index (BMI) [4]. In recent years, A Body Shape Index (ABSI) has emerged as a novel anthropometric measurement, which is suggested to be a superior predictor compared to BMI in many aspects [4,5]. Body fat percentage (BF%), another crucial anthropometric parameter, tends to increase with age and is considered one of the most significant indicators of metabolic changes in older adults [6]. However, there are only a limited number of studies investigating the relationship between ABSI, BF%, and vitamin D levels.

The aim of this study is to investigate whether there is an association between comprehensive geriatric assessment results, anthropometric measurements (BMI, ABSI, BF%), and vitamin D levels in elderly individuals.

## Methods

### Study design

This study was designed as a retrospective cross-sectional analytical study. The medical records of patients aged 65–100 who were enrolled in the Home Healthcare Services (HHS) of Izmir City Hospital and received home visits between December 2023 and December 2024 were reviewed.

To ensure statistical power and generalizability, we defined our sample size target as the maximum number of patients meeting the inclusion criteria and having documented vitamin D levels within the study period.

Data extracted from patient records included sociodemographic and clinical variables such as age, gender, and existing diseases, as well as anthropometric measurements (height, weight, waist circumference, and skinfold caliper measurements at the triceps, biceps, subscapular, and suprailiac regions) and detailed geriatric assessment results (activities of daily living, nutritional status, mental state, depression, frailty, and sarcopenia). Chronic diseases such as diabetes mellitus, hypertension, dementia, cerebrovascular disease, coronary artery disease, renal failure, and cancer were identified based on pre-existing diagnoses documented in the patients’ electronic medical records, confirmed through ICD-10 codes and ongoing medication records in the home healthcare database.BMI, ABSI, and body fat percentage (BF%) were evaluated as continuous variables.

The study has a retrospective design and was conducted on medical records. The data for research purposes were accessed between 14/02/2025 and 24/02/2025. All data were evaluated by being completely anonymized before being accessed by the researchers. The study was approved by the Izmir City Hospital Non-Interventional Ethics Committee with the decision number 2025/67 dated 13.02.2025.

Patients were excluded from the study if they:

- Were unable to communicate,
- Had incomplete questionnaire responses,
- Were unable to maintain a sitting or upright position for anthropometric measurements,
- Had a diagnosis of schizoaffective disorder,
- Had an active cancer diagnosis, or
- Had experienced trauma, cardiovascular disease, or cerebrovascular disease within one month before the examination.

### Comprehensive geriatric assessment tests

#### Barthel index.

This scale measures an individual’s ability to perform activities of daily living (ADL). The assessed activities include eating, bathing, personal hygiene, dressing, bowel and bladder control, toilet use, bed-to-wheelchair transfer, mobility, and stair climbing. Higher scores indicate greater independence in daily life. The Turkish version of the index has undergone validity and reliability testing [7].

#### Mini nutritional assessment-short form (MNA-SF).

This form, adapted and validated in Turkish, evaluates individuals’ nutritional status. The scoring ranges from 0 to 14, where; 12–14 points indicate normal nutritional status, 8–11 points suggest a risk of malnutrition, ≤ 7 points indicate the presence of malnutrition [8].

#### Mini-mental state examination (MMSE).

This test assesses cognitive function, with scores ranging between 0 and 30. Lower scores indicate worsening cognitive impairment, with severe cases suggesting dementia. The Turkish version of the test has been validated [9].

#### Geriatric depression scale-15 (GDS-15).

This scale evaluates depressive symptoms in elderly patients, assessing factors such as energy levels, enjoyment of life, hopelessness, loneliness, anxiety, and feelings of worthlessness. The 15-item self-report scale is scored from 0 to 15, where; 0–4 points indicate normal mood, 5–8 points suggest mild depression, 9–11 points indicate moderate depression, 12–15 points suggest severe depression.

The Turkish version of the scale has been validated for reliability [10].

#### Clinical frailty scale (CFS).

This scale assesses frailty levels based on a clinician’s observations, with scores ranging from 1 to 9. A score of 1 represents a very fit individual and a score of 9 indicates a patient in the terminal stage of life.

Scores ≥5 are considered indicators of frailty. The Turkish version of the scale has been validated [11].

#### Strength, assistance with walking, rising from a chair, climbing stairs, and falls (SARC-F) questionnaire.

This tool is used for rapid screening of sarcopenia, assessing muscle mass loss and functional decline. The scale is scored from 0 to 10, where scores of 4 or higher indicate the presence of sarcopenia. The Turkish version of SARC-F has been validated for reliability [12].

Pressure ulcers were evaluated by family physicians during home visits through direct clinical observation and patient records, and classified according to NPIAP staging criteria. The presence of a pressure ulcer was recorded as a binary (yes/no) variable.

### Anthropometric measurements

Anthropometric measurements, including height (m), weight (kg), waist circumference (WC) (m), and skinfold thickness, were performed by trained healthcare professionals following standardized procedures and using calibrated instruments. Height (m) was measured to the nearest 0.1 cm using a stadiometer (SECA 213 Portable Stadiometer, Hamburg, Germany) with participants standing barefoot, heels together, arms at sides, and head in the Frankfurt horizontal plane. Weight (kg) was measured to the nearest 0.1 kg using a portable digital scale (SECA 877, Hamburg, Germany) with participants wearing light clothing and no shoes. Waist circumference (WC) (m) was measured with a non-stretchable fiberglass tape (SECA 201, Hamburg, Germany) to the nearest 0.1 cm at the midpoint between the lower margin of the last palpable rib and the top of the iliac crest, with the participant standing and at the end of a normal expiration.Body Mass Index (BMI) was calculated using the standard formula:


$$BMI=weight\;(kg)/height^{2}\;(m^{2})\mathrm{\backslash ]}$$

A Body Shape Index (ABSI) was calculated using the following formula [4]:


ABSI=WC (m)/[BMI ^ (2/3)×height^(1/2) (m)].\]

Skinfold thickness was measured using a Harpenden Lange Skinfold Caliper (Cambridge Scientific Industries, INC., Cambridge, Maryland, USA) with constant spring pressure (10 g/mm²). Four anatomical sites on the right side of the body were measured based on the Durnin and Womersley protocol [13]:

Triceps: vertical fold at the midpoint between the acromion and olecranon processes on the posterior upper arm,
Biceps: vertical fold at the midpoint on the anterior aspect of the upper arm,
Subscapular: oblique fold located 1–2 cm below the inferior angle of the scapula,
Suprailiac: oblique fold just above the iliac crest along the midaxillary line.

Participants were in a relaxed standing or seated position, and skinfolds were measured three times at each site by the same examiner to reduce inter-observer variability. The mean of the three readings was used in all analyses.

Body density was estimated using the Durnin and Womersley equation specific to age and sex [13]. Subsequently, body fat percentage (BF%) was derived from body density using the Siri equation:


BF%=[(4.95/ body density)−4.50]×100\]

All instruments were calibrated prior to use, and standardized measurement conditions were maintained throughout the study.

### Vitamin D measurement results

Serum 25(OH)D levels were measured in the Biochemistry Laboratory of İzmir City Hospital using the electrochemiluminescence immunoassay (ECLIA) method on the Roche Cobas e601 analyzer. All analyses were performed under ISO 15189 accreditation, following the manufacturer’s instructions and daily internal quality control procedures. We have adopted the classification proposed in a recent international consensus statement on vitamin D in older adults, which defines serum 25(OH)D levels as follows [3,14]:

- Severe deficiency: < 10 ng/mL
- Deficiency: 10–19 ng/mL
- Sufficient: ≥ 20 ng/mL

### Statistical analysis

The data were analyzed using the Statistical Package for the Social Sciences (SPSS) version 26.0. Descriptive characteristics of the patients are presented as numbers and percentages. All numerical data are provided as mean, standard deviation, median, minimum, and maximum. The normality of the data distribution was checked using the Kolmogorov-Smirnov test, and it was found that the data did not follow a normal distribution. Therefore, the comparison of numerical (continuous) data was conducted using the Kruskal-Wallis H Test. The comparison of categorical parameters was performed using the Chi-Square Test. Post Hoc Tests were used to determine differences between groups. The relationships between vitamin D levels and all other variables were examined using Spearman’s Correlation Analysis. The effects of vitamin D levels on ABSI score and the presence of pressure ulcers were analyzed through linear regression analysis. Significance levels of 0.05 and 0.01 were considered for the entire study.

## Results

A total of 439 patients aged 65–100 years who met the inclusion criteria and had complete data were included in the study. In univariate statistics, all variables were analyzed according to individuals’ vitamin D levels. When examining patients’ sociodemographic characteristics and chronic disease status, no significant relationships were found between vitamin D and other variables, except for hypertension (p = 0.049) (p > 0.05) (Table 1). ABSI values were higher in individuals vitamin D level with severe deficient and deficient compared to other groups, while BMI levels were found to be higher in the vitamin D deficient group (p < 0.001, p < 0.001, respectively) (Table 2). Additionally, individuals in the severe vitamin D deficiency group had a higher frequency of pressure ulcers and lower Barthel Index scores (p = 0.033 and p = 0.026, respectively) (Table 3).

**Table 1:** Comparison of sociodemographic characteristics and chronic diseases by vitamin D levels.

*Variables*		Severe Deficiency (<10 ng/mL)			Deficiency (10–20 ng/mL)		Sufficient (≥20 ng/mL)		*p*
		*n*		*%*	*n*	*%*	*N*	*%*	
Gender	Male	43		36.4	86	44.6	46	35.9	0.204
	Female	75		63.6	107	55.4	82	64.1	
Diabetes Mellitus	No	77		65.3	131	67.9	79	61.7	0.525
	Yes	41		34.7	62	32.1	49	38.3	
Hypertension	No	41		34.7	93	48.2	50	39.1	0.049*
	Yes	77		65.3**a**	100	51.8**b**	78	60.9**c**	
Coronary Artery Disease	No	86		72.9	155	80.3	89	69.5	0.073
	Yes	32		27.1	38	19.7	39	30.5	
Renal Failure	No	110		93.2	179	92.7	114	89.1	0.403
	Yes	8		6.8	14	7.3	14	10.9	
Cancer	No	106		89.8	178	92.2	112	87.5	0.373
	Yes	12		10.2	15	7.8	16	12.5	
Dementia	No	104		88.1	181	93.8	110	85.9	0.053
	Yes	14		11.9	12	6.2	18	14.1	
Cerebrovascular Event	No	99		83.9	173	89.6	106	82.8	0.161
	Yes	19		16.1	20	10.4	22	17.2	
			*Mean ± SD* *Median (Min-Max)*		*Mean ± SD* *Median (Min-Max)*		*Mean ± SD* *Median (Min-Max)*		*P*
Age^H^		80.27 ± 8.23 80 (65 - 96)			79.78 ± 8.22 81 (65 - 99)		81.57 ± 7.60 82 (65 - 98)		0.146

*p < 0.05; **p < 0.01, Categorical variables: Chi-square test, H: Kruskal Wallis H Test, Lettering: Different letters indicate the difference within the groups (Duncan’s Multiple Comparison Test).

**Table 2:** Comparison of anthropometric measurements by vitamin D levels.

*Variables*	Severe Deficiency (<10 ng/mL)	Deficiency (10–20 ng/mL)	Sufficient (≥20 ng/mL)	*p*
	*Mean ± SD* *Median (Min-Max)*	*Mean ± SD* *Median (Min-Max)*	*Mean ± SD* *Median (Min-Max)*	*p*
BMI^H^	25.09 ± 4.60 24.35 (14.7–35.6)**a**	28.77 ± 8.11 26.2 (19.5–76.02)**b**	25.92 ± 7.65 25.35 (13.1–54.7)**a**	0.000**
ABSI^H^	0.128 ± 0.022 0.121 (0.094–0.226)**b**	0.128 ± 0.022 0.124 (0.096–0.198)**b**	0.118 ± 0.017 0.114 (0.085–0.171)**a**	0.000**
Body Fat Percentage^H^	33.58 ± 5.09 33.79 (18.47–42.76)	33.31 ± 5.16 33.78 (19.68–50.54)	33.66 ± 4.87 34.16 (20.06–43.06)	0.808

*p < 0.05; **p < 0.01, H: Kruskal Wallis H Test, Lettering: Different letters indicate the difference within the groups (Duncan’s Multiple Comparison Test), BMI: Body Mass Index, ABSI: A Body Shape Index.

**Table 3:** Comparison of geriatric assessment results by vitamin D levels.

*Geriatric Assessment Variables*		Severe Deficiency (<10 ng/mL)		Deficiency (10–20 ng/mL)		Sufficient (≥20 ng/mL)		*P*
		*n*	*%*	*N*	*%*	*n*	*%*	
ONS	No	88	74.6	157	81.3	103	80.5	0.333
	Yes	30	25.4	36	18.7	25	19.5	
Pressure Ulcer	No	34**a**	28.8	80**ab**	41.5	56**b**	43.8	0.033*
	Yes	84**a**	71.2	113**ab**	58.5	72**b**	56.3	
MNA-SF	Normal	29	24.6	64	33.2	36	28.1	0.496
	Malnutrition risk	54	45.8	82	42.5	61	47.7	
	Malnutrition	35	29.7	47	24.4	31	24.2	
GDS-15	No	46	39.0	100	51.8	51	39.8	0.102
	Mild	40	33.9	63	32.6	52	40.6	
	Moderate	15	12.7	16	8.3	11	8.6	
	Severe	17	14.4	14	7.3	14	10.9	
SARC-F	Below 4	9	7.6	13	6.7	10	7.8	0.923
	4 and above	109	92.4	180	93.3	118	92.2	
CFS	Below 5	27	22.9	62	32.1	43	33.6	0.132
	5 and above	91	77.1	131	67.9	85	66.4	
		*Mean ± SD* *Median (Min-Max)*		*Mean ± SD* *Median (Min-Max)*		*Mean ± SD* *Median (Min-Max)*		*P*
Barthel Index^H^		46.19 ± 24.65 50 (0–100)**a**		53.95 ± 25.45 50 (0–100)**b**		48.22 ± 2528 50 (0–100)**ab**		0.026*
MMSE^H^		13.26 ± 9.51 13 (0 - 30)		13.98 ± 9.04 15 (0 - 30)		14.29 ± 8.72 15 (0 - 28)		0.690

*p < 0.05; **p < 0.01, H: Kruskal Wallis H Test, Categorical variables: Chi-square test. Lettering: Different letters indicate the difference within the groups (Duncan’s Multiple Comparison Test), ONS: Oral Nutrisyon Solüsyonu, MNA-SF: Mini nutritional assessment short form, GDS-15: Geriatric depression scale-15, SARC-F: Strength, assistance with walking, rising from a chair, climbing stairs, and falls questionnaire, CFS: Clinical Frailty Scale, MMSE: Mini-mental status test.

When the relationships between individuals’ vitamin D levels and all variables were examined using correlation analysis, a statistically significant negative relationship was found between vitamin D levels and ABSI (r = −0.184, p < 0.05) and the presence of pressure ulcers (r = −0.113, p < 0.05) (Table 4). In the regression analysis results, a negative and significant relationship was found between vitamin D levels and ABSI score (β = −0.179, p < 0.05) and the presence of pressure ulcers (β = −0.113, p < 0.05) (Table 5). Hypertension and BMI, which were found to have a significant relationship with vitamin D in univariate statistics, lost their significance in correlation and regression analyses.

**Table 4:** Correlation analysis between vitamin D levels and clinical characteristics.

*Variable*	*Coefficient*	*Vitamin D level*	*Variable*	*Coefficient*	*Vitamin D level*
Gender	r	0.007	Dementia	R	0.032
	p	0.883		p	0.510
Age	r	0.066	Cerebrovascular Event	r	0.015
	p	0.165		p	0.752
BMI	r	0.008	ONS	r	−0.053
	p	0.875		p	0.271
ABSI	r	**−.184**	Barthel Index	r	0.026
	p	0.000**		p	0.593
Body Fat Percentage	r	−0.003	MNA-SF	r	−0.043
	p	0.954		p	0.372
Diabetes Mellitus	r	0.030	MMSE	r	0.039
	p	0.537		p	0.415
Hypertension	r	−0.028	CFS	r	−0.084
	p	0.552		p	0.072
Coronary Artery Disease	r	0.033	SARC-F	r	−0.003
	p	0.491		p	0.944
Renal Failure	r	0.058	GDS-15	r	−0.028
	p	0.227		p	0.554
Cancer	r	0.032	Pressure Ulcer	r	**−.113**
	p	0.510		p	0.018*

*p < 0.05; **p < 0.01. BMI: Body Mass Index, ABSI: A Body Shape Index, ONS: Oral Nutrisyon Solüsyonu, MNA-SF: Mini nutritional assessment short form, MMSE: Mini-mental status test, CFS: Clinical Frailty Scale, SARC-F: Strength, assistance with walking, rising from a chair, climbing stairs, and falls questionnaire, GDS-15: Geriatric depression scale-15.

**Table 5:** Regression analysis results for the effects of vitamin D levels on ABSI scores and pressure ulcers.

Dependent Variable	Path	Independent Variables	*B*	*S.E.*	*β (Beta)*	*p*	*R* ^*2*^
ABSI	<---	Vitamin D level	−6.236	1.637	−0.179	0.000**	0.179
Pressure Ulcer	<---	Vitamin D level	−0.174	0.073	−0.113	0.018*	0.113

B: Unstandardized path coefficient, β (Beta): Standardized path coefficient, *p < 0.05, **p < 0.01, Variance ratio explained in dependent factors: R2, ABSI: A Body Shape Index.

## Discussion

In this study, we investigated the relationship between vitamin D levels and the demographic, geriatric, and anthropometric characteristics of patients aged 65 and older receiving home healthcare services. The correlation and regression analysis results indicated a negative association between vitamin D levels and both pressure ulcer presence and ABSI values.

In our study, the mean ABSI score was calculated as 0.12 ± 0.02. While the typical ABSI reference range in the general adult population is reported to fall between 0.07 and 0.09, previous research has indicated that ABSI values tend to rise with increasing age. For example, a large-scale study involving 944,769 individuals aged 65 and older reported mean ABSI scores of ≥0.10, supporting this trend [15]. Similarly, Zhang et al. observed ABSI values above 0.10 in certain elderly participants [16], and another study by the same group further confirmed that such values are not uncommon among older adults [17]. This elevation is believed to result from age-related alterations in body composition, such as a decline in muscle mass and a relative increase in central adiposity, both of which are effectively captured by ABSI.

Considering that the aging population worldwide has a higher BMI, it can be hypothesized that the increased fat mass leads to a larger distribution volume for vitamin D, a fat-soluble vitamin [3]. While obesity is increasingly recognized as a key factor in low vitamin D levels, BMI alone is insufficient to reflect overall obesity and, more importantly, central body fat accumulation [18]. Since ABSI incorporates both BMI and waist circumference, it has been suggested as a better predictor of obesity and central body fat [19]. In our study, a significant negative relationship was also found between ABSI score and vitamin D levels. However, BMI, which was associated with vitamin D deficiency in univariate statistics, lost its significance in correlation and regression analyses. Limited studies in the literature support this finding. In a study by Ren et al., which examined data from 1,666 patients over 18 years old, an inverse relationship between ABSI and vitamin D levels was found, consistent with our findings [4]. Similarly, Patriota et al. and Zhu et al. found a significant negative relationship between ABSI and serum vitamin D levels in adult male populations [20,21]. However, there is a lack of sufficient studies examining the relationship between ABSI and vitamin D in geriatric populations. Given the age-related increase in body fat, it is plausible that vitamin D is trapped in adipose tissue, leading to lower serum levels. Therefore, ABSI, a strong indicator of central obesity and fat accumulation, may also serve as a useful predictor of vitamin D levels. Supporting this interpretation, a recent large-scale cohort study by Du et al. involving over 8,000 older adults demonstrated that higher ABSI score was significantly associated with impaired ADL. Their findings suggest that these novel anthropometric indices are not only linked to metabolic disturbances but also serve as predictors of functional decline in aging populations. This aligns with our findings, in which ABSI was negatively associated with vitamin D levels—an essential factor known to affect musculoskeletal health and physical function. Taken together, these results highlight ABSI as a promising integrated marker that may reflect both biological and functional vulnerabilities in older adults [22]

Efficient wound healing is crucial for protecting the body from fluid loss and infections. Vitamin D and calcium play essential roles in regulating these functions in the skin. Vitamin D deficiency has been observed in patients with chronic, slow-healing wounds, and topical vitamin D applications have been shown to aid wound healing [23]. A 2024 Cochrane review on nutritional interventions for the prevention and treatment of pressure ulcers highlighted the limited number of studies investigating the relationship between vitamin D levels and pressure ulcers. Although evidence remains scarce, the review suggested that a standard diet enriched with vitamin D may be effective in preventing pressure ulcer formation [24]. Similarly, Smith et al., in a meta-analysis of 10 studies involving 2,359 patients, concluded that individuals with chronic wounds tend to have lower vitamin D levels [25]. In another study, Wang et al. found that patients with diabetes who had lower vitamin D levels developed diabetic foot ulcers more rapidly and suggested that vitamin D supplementation might help protect these patients from diabetic foot ulcers [26]. Vitamin D may positively impact the wound healing process by enhancing immune response, promoting fibroblast and keratinocyte proliferation, and supporting differentiation [23]. To better understand the relationship between vitamin D levels and pressure ulcers, future studies should focus specifically on patient groups with pressure ulcers. Additionally, monitoring serum vitamin D levels and implementing appropriate treatment strategies in patients with delayed wound healing could positively influence the healing process.

In our univariate analysis, we found that patients with hypertension were more likely to have severe vitamin D deficiency; however, this relationship lost significance in multivariate analyses. Experimental studies suggest that endothelial dysfunction may contribute to the development of hypertension and that vitamin D plays a role in regulating endothelial function [27]. Additionally, vitamin D has been found to reduce the activity of the renin-angiotensin-aldosterone system (RAAS), leading to vasodilation and a potential protective effect against hypertension [28]. The National Health and Nutrition Examination Survey (NHANES), a major U.S. national dataset, found a non-linear relationship between serum vitamin D concentration and blood pressure levels [29]. However, the same study reported that when vitamin D levels exceeded 84 nmol/L, diastolic blood pressure increased, suggesting the need for further research to clarify this relationship [29]. A systematic review by Adamczak et al. on the association between vitamin D and hypertension concluded that while experimental and observational studies support a role for vitamin D in hypertension pathogenesis, prospective clinical trials have not demonstrated a significant antihypertensive effect of vitamin D supplementation [28]. Similarly, a prospective cohort study by Sheehy et al., conducted on Black women, found a weak inverse relationship between vitamin D levels and hypertension incidence. The authors suggested that this weak association may be influenced by confounding factors that were not fully controlled [30]. Overall, studies at lower levels of the evidence hierarchy suggest a significant association between vitamin D levels and hypertension, but as the quality of evidence increases, this relationship weakens. Considering the vascular changes occurring in geriatric patients, conducting age- and disease-specific studies could help provide further clarity on this issue.

Although the relationship between dementia and vitamin D levels did not reach statistical significance (p = 0.053), the near-threshold value may indicate a potential trend, consistent with previous studies suggesting that vitamin D deficiency may contribute to cognitive decline in older adults [31,32].

Our initial analyses showed that as daily living activity levels decreased, vitamin D levels also declined; however, this association lost statistical significance in multivariate models. A systematic review by Fleer et al. on stroke patients found that vitamin D supplementation improved mobility but did not significantly impact daily living activities. They also emphasized the need for randomized controlled trials (RCTs) to confirm these findings [33]. A study by Zhang et al., conducted on 943 patients aged over 100 years, found a significant positive association between vitamin D levels and daily living activities [34]. Similarly, Sahin Alak et al., in their study of 370 geriatric outpatients, also found a significant positive relationship between daily living activities and vitamin D levels [14]. However, in contrast, a randomized controlled trial by Bischoff-Ferrari et al., which followed 2,157 patients aged 70+ over three years, found that vitamin D supplementation did not impact patients’ physical performance [35]. Since vitamin D deficiency can manifest as muscle pain and weakness, it may negatively impact patients’ mobility and daily activities. However, various other factors also influence functional independence in older adults. Thus, in elderly individuals with reduced mobility, assessing and correcting deficiencies not only in vitamin D but also in other essential nutrients should be prioritized.

### Limitations

Due to the retrospective cross-sectional analytical design of our study, it is not possible to determine whether the negative association between vitamin D levels, ABSI, and pressure ulcers is causal or merely correlational. Conducting longitudinal studies on individuals aged 65 and older could help clarify this relationship. Additionally, the fact that this study was conducted on patients requiring home healthcare services presents a limitation. Including more active elderly individuals and accessing a broader segment of the geriatric population could provide a more comprehensive understanding of the subject.The retrospective design of the study did not allow for adjustment of seasonal variation, although all measurements were conducted during months with relatively higher sunlight exposure.

Also some potential confounders such as physical activity, vitamin D supplementation, smoking, and seasonality could not be included in the analysis due to missing or unavailable data in patient records. However, the sample was homogeneous in terms of age, gender, BMI, and chronic conditions.

## Conclusion

In this study, lower vitamin D levels were associated with higher ABSI scores and increased prevalence of pressure ulcers in elderly home care patients. While BMI and hypertension initially showed associations with vitamin D, these were not sustained in multivariate analyses. ABSI may serve as a more precise anthropometric indicator for detecting vitamin D deficiency in older adults. Additionally, regular vitamin D screening is recommended in patients with pressure ulcers to support healing and improve outcomes.

## Supporting information

S1 Data Database. (SAV)
